# Investigating Non-Visual Eye Movements Non-Intrusively: Comparing Manual and Automatic Annotation Styles

**DOI:** 10.16910/jemr.15.2.1

**Published:** 2022-04-22

**Authors:** Jeremias Stüber, Lina Junctorius, Annette Hohenberger

**Affiliations:** University of Osnabrück, Germany

**Keywords:** Eye movement, non-visual, gaze, saccades, annotation, usability

## Abstract

Non-visual eye-movements (NVEMs) are eye movements that do not serve the provision of visual information. As of yet, their cognitive origins and meaning remain under-explored in eye-movement research. The first problem presenting itself in pursuit of their study is one of annotation: in virtue of their being non-visual, they are not necessarily bound to a specific surface or object of interest, rendering conventional eye-trackers nonideal for their study. This, however, makes it potentially viable to investigate them without requiring high resolution data. In this report, we present two approaches to annotating NVEM data – one of them grid-based, involving manual annotation in ELAN (18), the other one Cartesian coordinate-based, derived algorithmically through OpenFace (1). We evaluated a) the two approaches in themselves, e.g. in terms of consistency, as well as b) their compatibility, i.e. the possibilities of mapping one to the other. In the case of a), we found good overall consistency in both approaches, in the case of b), there is evidence for the eventual possibility of mapping the OpenFace gaze estimations onto the manual coding grid.

## Introduction

Non-visual eye-movements (NVEMs) are eye-movements that do not serve the provision of visual information. Various theories exist as to their raison d’être, depending on the context of their production (8). For instance, during public or conversational speaking, they might serve to convey certain information about the interlocutor’s affective or cognitive state (9). A commonly held belief, purported for instance by adherents to neuro-linguistic programming (NLP), is that certain eye gaze directions are indicative of “remembering, imagining, or having an internal dialog” (9). These claims point towards an area of research that has not yet been explored in much detail, nor with methodological rigor. However, see Diamantopoulos et al. (6) for a critical review on past research.

A basic difficulty that arises in wanting to investigate correlations between cognitive processes and NVEMs is one of annotation. In regular eye-tracking tasks designed around eye-movements, it is appropriate to constrain their capture to an area of visual interest, such as a screen on which visual stimuli appear. In the case of NVEMs, however, eye gazes are not bound to a specific area of visual interest, necessitating their capture in as wide a range as possible. Since the captured area of conventional *remote*, or *head-stabilizing*, eye-tracking devices is generally limited to the screen on which stimuli appear, but NVEMs are liable to fall outside of this area, these devices are unfit to the task the task requirements (see 4, p. 50). Furthermore, by limiting participants’ freedom of movement through the employment of head-stabilizing equipment, NVEM behavior may be altered. While wearable devices are less constraining in this regard, they naturally alert the participant as to the research’s focus on eye movements, thereby also increasing the chances of altering their NVEM behavior during trials. However, in light of the non-visual nature of NVEMs, it is unclear whether high resolution capture is even necessary in the first place, which opens up the possibility to apply less precise yet equally valid methodologies to their study.

**Figure 1.**
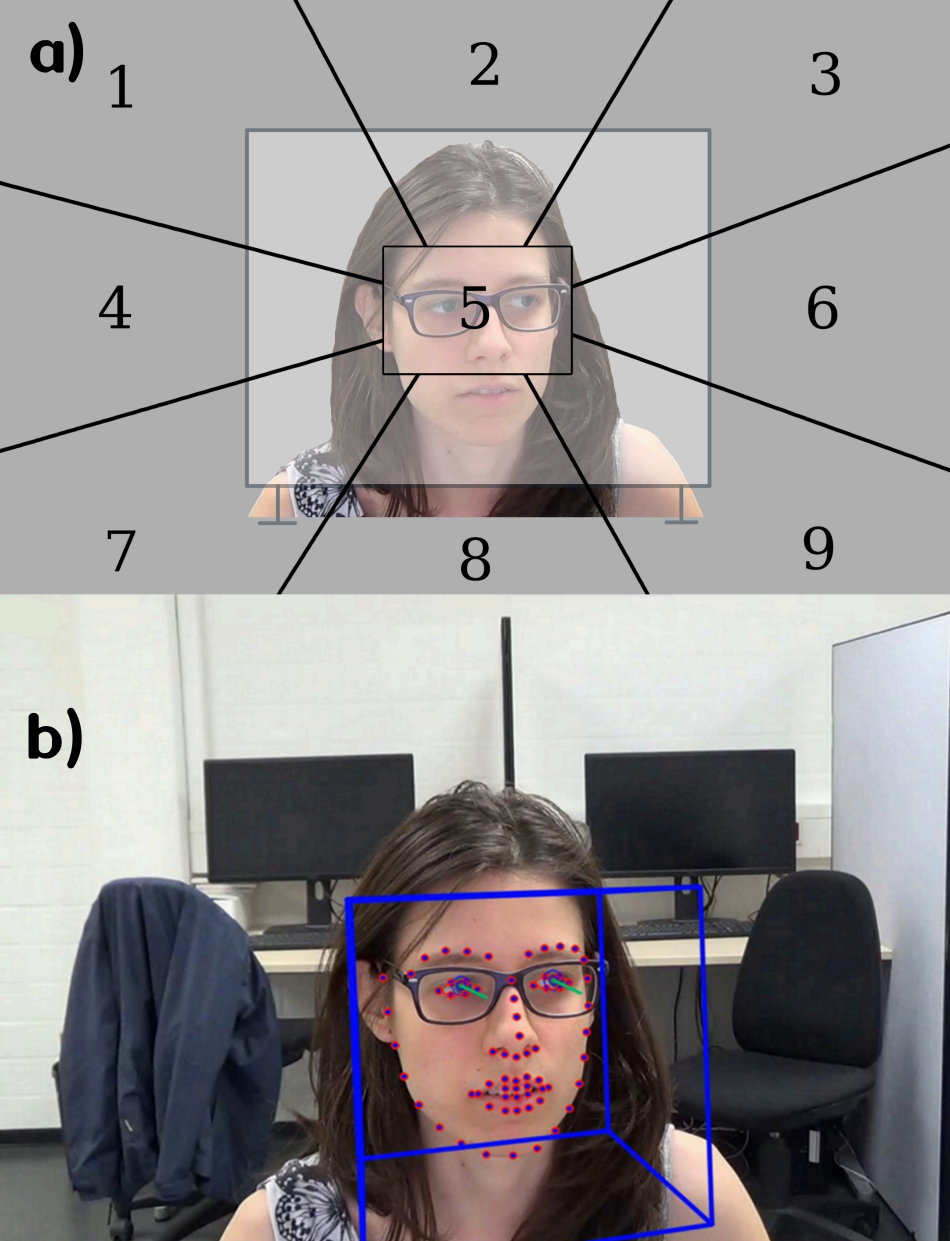
a) Coding grid used for the manual annotation. The inclusion of the translucent screen in the image depicts the non-bounded spatial extension of the radial grid sections. During annotation, the grid was mentally super-imposed upon the video footage. This grid was super-imposed upon the screen during annotation. Since the participants were facing the camera during the recording process, their gazes appeared mirrored. In other words, a leftwards gaze from the viewpoint of the participant was annotated as ‘6’, which is found on the right-hand side of the coding grid. Similarly, a participant gazing towards the lower right side was annotated as ‘7’. Note that there was a tenth gaze direction ‘0’ which was used whenever participants looked into the camera or closed their eyes for a longer period. b) Visual output of OpenFace. The blue box represents the head pose position. The dots represent the landmarks, and the green lines represent the eye-gaze vectors which were used in our analysis.

Here, we present and discuss two approaches that attempt to enable the non-intrusive study of NVEMs. Both approaches operate on the basis of face-centered video footage of participants, obtained from an external camera positioned above the computer screen. The first approach involves manual annotation in the open-source application *ELAN* (18) according to a coding grid which divides the visual field into nine sections, as seen in Fig. 1. The second approach makes use of the open-source neural-network driven face-recognition software *OpenFace 2.0* (1), allowing for the representation of the participant’s NVEMs in terms of Cartesian vectors. Our aim will be a) to evaluate whether each of the two approaches is at all suitable for the study of NVEMs, and b) if they are, in which specific research conditions one is preferable over the other. We found that our evaluation of one approach was in fact complemented by the respective other by providing supplementary information. Thus, our analyses also offer insight into the potential compatibility of the approaches.

## Experimental Methods

**Figure 2.**
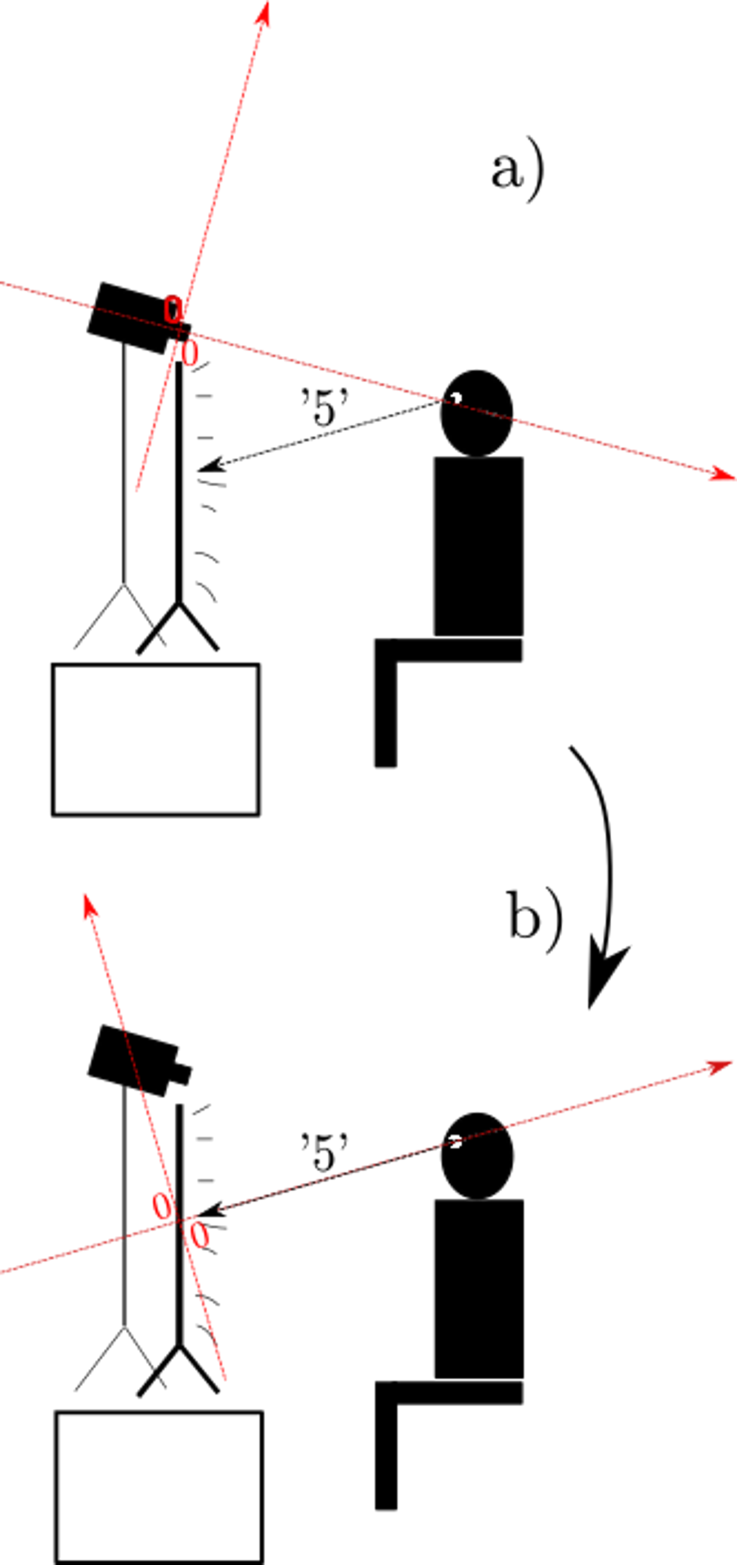
a) Experiment set-up in two dimensions for ease of representation; what is depicted here as a rotation around only one axis, was in reality a rotation around two axes. The coordinate system in red is the original coordinate system generated by OpenFace during the automatic annotation. The vector originating from the participant is the ‘5’ vector, i.e. the one that aimed at the center of the screen. b) After rotating the coordinate system, its origin is now in the center of the screen and oriented such that the x-axis coincides with the ‘5’ vector.

The video material annotated and analyzed here, had been collected in the scope of a wider project, namely to investigate whether NVEMs differ in terms of (past and future) episodic and semantic cognition. Refer to Fig. 2 for a view of the experimental setup. Since we are only interested in the evaluation of different annotation methods here, we do not further elaborate on the content of that project but refer the reader to Appendix 1 where we explain the experimental method and rationale in more detail.

### Manual Annotation with Coding Grid

Our first approach to analyze the collected data was by annotating it manually. We used the open-source software ELAN which can display a video and an audio track simultaneously. The annotation is performed with the aid of tiers which are layers of text linked to selected intervals in the video and audio track. In addition to the linguistic utterances, and the division of the trials into question and answer, we coded the participant’s gaze directions for each trial. To make the process more reliable and to facilitate it, we used a coding grid dividing the participant’s field of vision into nine sections (see Fig. 1 a)). The sections are arranged in a 3 by 3 design with one center area being framed by eight radial segments. For easy referencing, the sections were numbered starting at the top left and proceeding line by line from left to right. Therefore, for example on the left side, the upper section is labeled ‘1’, the middle section is labeled ‘4’, and the lower section is labeled ‘7’. The center of the screen was labeled '5'. Here, an image of the object for which participants had to narrate a (past or future) episode or give a semantic description, was displayed. This effectively rendered the central section (‘5’) a natural and recurrent reference point for the participants’ gazes because it was directly in front of them and they were primed to return to it at the beginning of each new trial. It is important to note that all sections apart from the central one are not bounded, in the sense that they were imagined by the annotators to extend beyond the edges of the screen (see Fig. 1). In other words, gaze directionality was encoded from a central point of reference (section '5'). Since the gazes were annotated from the camera perspective, the gaze directions are vertically mirrored in relation to the participant’s point of view. A tenth gaze direction ‘0’ was used in case the gaze could not be interpreted because a participant looked directly into the camera or closed their eyes for longer than a blink.

**Figure 3.**
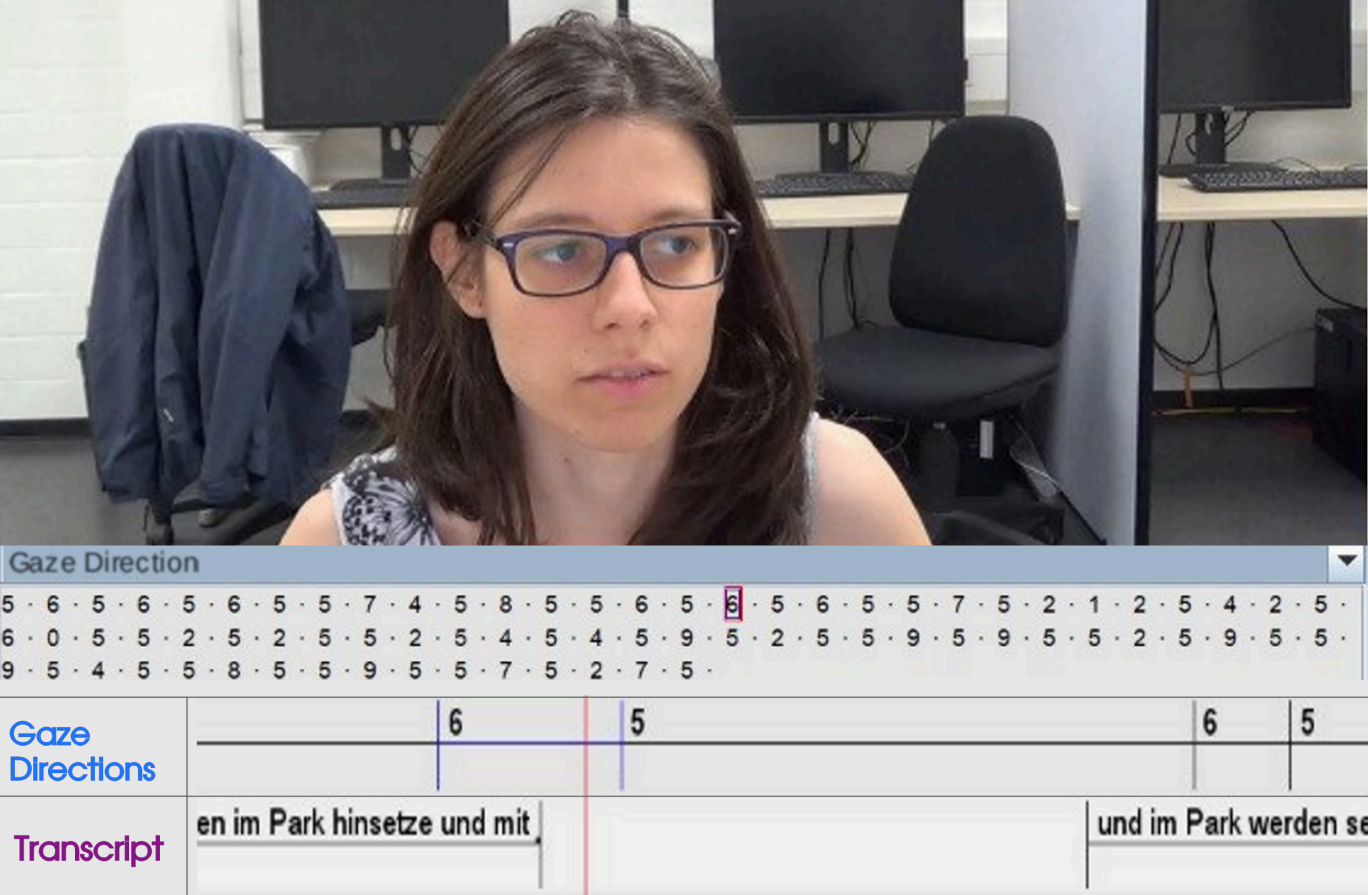
Modified screenshot of the annotated video and audio track of one participant in the software ELAN. At the top, the video can be seen. At the bottom, annotation tiers for the gaze direction and the transcription are shown. The participant’s eye gaze in the displayed moment was labeled ‘6’ as seen in the tier “Gaze Directions” (see also Fig. 1 a)). Between the video and the tiers, the sequence of all gaze directions annotated for this participant can be found. This is not to be confused with a scanpath, as it merely represents a temporal progression of categories.

To annotate, the raters went over each trial creating intervals representing the length of each gaze and assigning the number of the grid section the respective gaze is directed to (‘0’ to ‘9’) (see Fig. 3). The beginning of an interval was set on the point in time the gaze started to move towards a new grid section in which it would rest next. Immediately before the gaze direction changed into a new grid section, i.e. at the end of a fixation period, the interval was terminated. Each interval therefore consists of the transition movement of the eye gaze from one grid section to another and its fixation in the new section. Please note that the annotation process relied on the anticipation of the raters who could access a complete view of the eye gaze progress instead of simply a sequence of snapshots. The raters annotated each gaze by watching and rewinding the respective part of the video. It allowed them to take into account where a gaze would land next, and made the goal-directed labeling of each gaze possible. Also, it is important to keep in mind that the raters interpreted all gazes with regards to the nine grid sections. At first, the grid printed on a transparent sheet was placed on the computer screen to train the rater in detecting the correct sections. Overall, the participants’ gazes were clearly identifiable. However, there was a blending of gaze directions towards the grid sections ‘4’ and ‘7’, as well as ‘6’ and ‘9’. Many gazes were on the shared border of these grid sections leading to ambiguous assignment and thus creating two mid-to-low peripheral sections.

The two raters worked separately on individual parts of the data. To validate their annotations, 20% of the trials of each participant, chosen at random, were additionally rated by the respective other rater. For these additional annotations, the respective second rater applied the same annotation procedure as described above but used the temporal intervals previously determined by the respective first rater. Using this 20% doubly annotated data, an interrater reliability test was performed.

The agreement of both annotations was determined with the aid of Cohen’s kappa computed with IBM SPSS Statistics 26 (results taken from Stelter (25)). The overall kappa value was 0.907. The lowest kappa value for the individual trials of all participants was 0.741 while the highest was 1.000. The lowest kappa value for the individual trials was 0.400 (this trial, however, only consisted of three gaze directions) while the highest was again 1.000. According to Landis and Koch (17) the overall kappa values of both grids can be interpreted as almost perfect agreement. The method of coding the gaze directions manually using a coding grid is thus reliable and therefore justified (25).

### Automatic Annotation with OpenFace

The manual annotation using the coding grid was, by design, a simplified, intuitive approach to NVEM categorization, in large part due to the somewhat arbitrary arrangement of the gaze-directions themselves. Even though they divide up the visual field into intuitively reasonable sections, as seen in Fig. 1, the resulting arrangement may only seem reasonable superficially and preclude other ways of interpreting the data. Furthermore, the coding grid annotations did not lend themselves to finer-grained analyses offered e.g. by saccade and fixation detection algorithms. Lastly, the annotation process proved to be quite labor intensive. For all these above reasons, we decided to turn to a finer-grained, automatic approach to annotation, less prone to human biases.

At the base of the second approach was the analysis of the recorded video footage of the participants using the open source software OpenFace (1). OpenFace takes standard video as input and outputs a frame-by-frame vectorized analysis of any faces contained therein (see Fig. 1 b)). Fixing the camera lens as the origin of a 3-dimensional Cartesian coordinate system, OpenFace calculates metrics such as *head position*, *eye-gaze vectors*, *eye-gaze angles* for each eyeball, as well as the locations of various facial landmarks. For our analysis, we took into account only the eye-gaze vectors.

Since the experiment had not been designed with an OpenFace analysis in mind, we had to perform various pre-processing steps before the raw OpenFace data could be put to use and serve as a meaningful basis for comparison between the participants. Although all participants were seated on a chair fixed in its position relative to the computer screen, we initially did not account for the small differences in body posture, head position and tilt, as well as the exact position of the camera. Therefore, in order to place all of the OpenFace data for each participant in approximately the same Cartesian coordinate system, i.e. a coordinate system whose origin is located at approximately the same point in space across all participants, we had to perform a coordinate system rotation.

Since each participant looked at the center of the screen from a slightly different angle and position, rather than measure the distance from the camera lens to the presumed center of the screen with a measuring tape, we re-engineered its position algorithmically for each participant using the coding grid annotations.

Recall that in the coding grid (Fig. 1 a)), the gaze-direction ‘5’ was defined as a participant’s gaze toward the center of the screen. We exploited this, as well as the fact that each participant rested their gaze on the ‘5’ for an ample number of frames throughout the entire experiment session. This allowed us to reliably calculate the mean vector for all the frames during which the participant had been determined to be looking at the gaze-direction ‘5’. Under the assumption that the resulting vector would be an appropriate approximation of, not the absolute, but the participant’s idiosyncratic screen center, we rotated each coordinate system such that the mean ‘5’ vector’s polar coordinates were 0° on the *x*-axis, and 0° on the *y*-axis (see Fig. 2 b)).

**Figure 4.**
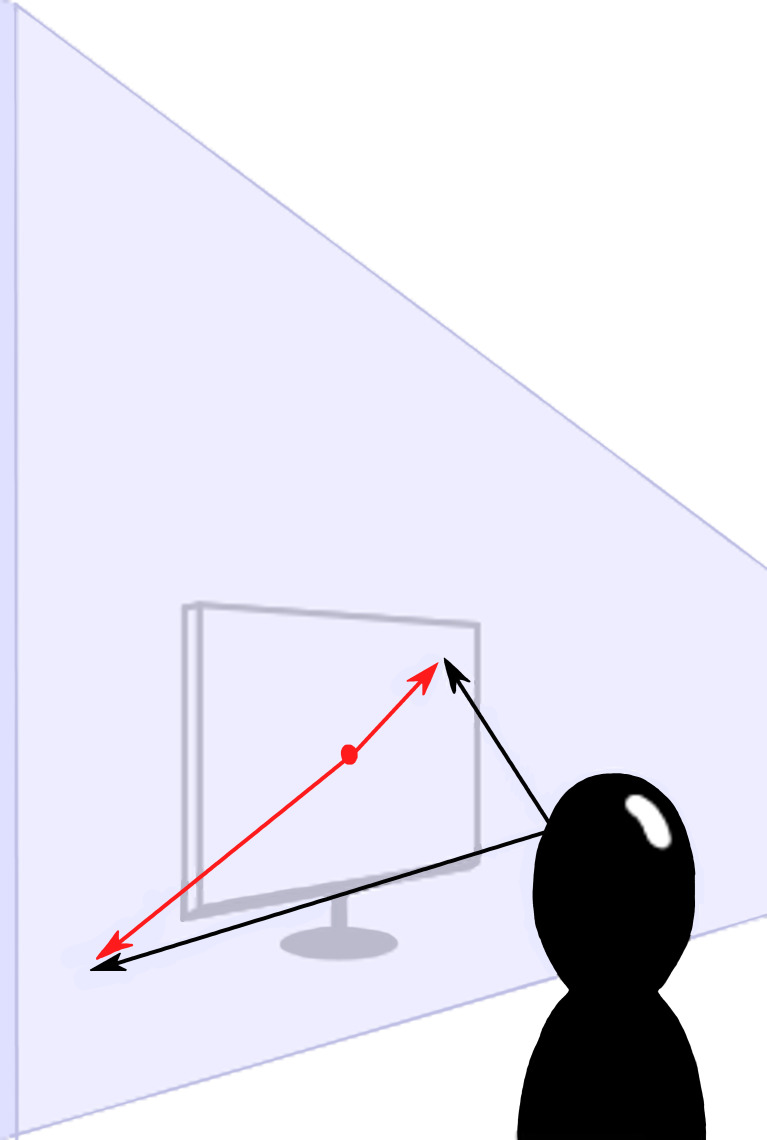
Vectors in black are the original 3-D coordinates generated by OpenFace, whereas the red vectors are the 2-D vectors resulting from the planar projection.

In a subsequent step, we decided to remove the depth dimension (*z*-axis in OpenFace) from our data by means of a planar projection, because this would allow us to employ eye-tracking algorithms in future analyses (see Fig. 4). Another reason for the removal was the fact that the depth dimension was ultimately irrelevant to our investigation, as NVEMs presumably do not have a well-defined focal point in three-dimensional space. Therefore, it would be impossible to estimate the depth at which the gaze focuses on a point or object. This assumption is supported by findings that show the occurrence of NVEMs even when one is alone in a physically barren environment (12; 19), in the dark (7), and even when one’s eyes are closed (8).

Effectively, we reduced the dimensions of the coordinates by one, converting 3-D coordinates into 2-D coordinates. As depicted in Fig. 4, the 2-D coordinates were located on a 2-dimensional plane in 3-D space. To achieve this, we had to determine a fixed depth away from the participants at which their gazes stopped. We chose a distance of 40 cm because that was the approximate distance from the center of the chair to the center of the screen. Then, we performed a planar projection onto the imaginary plane that extends perpendicular to the *x*-axis of the rotated coordinate system rotation from the previous step. In other words, after the planar projection, the resulting vectors were no longer represented in 3-dimensional space, but were instead located on that plane, described only by their newly calculated *x* and *y* vector components. The origin of this new, 2-D coordinate system was the same as that of the rotated 3-D coordinate system, as the origin of the latter already lay in the assumed center of the screen.

Upon plotting the 2-D vectors, we noticed that their range was more limited than we had expected, extending only ~20 cm in each direction both horizontally and vertically. This did not add up with our intuitive estimates, seeing as the screen itself is already 40 cm wide. With an average gaze span of only 40 cm, this would mean that the participants’ gazes rarely ventured beyond the edges of the screen, something that did not add up with what the recorded footage suggested. Since OpenFace provides video output in which the vectors it calculated are drawn into the original footage (see Fig. 1 b)) , we performed a visual side-by-side comparison between the eye gazes in the original videos and the OpenFace vectors.

Looking at individual frames in which the participant’s eye gaze was most extreme in its deflection from the center, we discovered that, in the OpenFace footage, the vectors consistently underestimated the gaze angle a human observer would expect (see Fig. 1, or Appendix 2). Other gazes directed in the general direction of the camera were for the most part captured accurately, but whenever a participant would gaze a little farther towards one side, horizontally or vertically, the vectors often did not accurately represent the full extent of the movement. In order to find out whether the source of the problem was in our camera set-up, we recorded new video footage similar to that of the participants, while making sure that the recording quality was as high as possible, the faces were properly lit, as well as calibrating OpenFace using the exact camera lens specifications. The problem persisted, however, and after some research on the matter, the performance we achieved seems to be expected, as “estimating gaze from webcam data is a really challenging problem overall” (T. Baltrušaitis, personal communication, May 28, 2021).

In order to assess whether this error was systematic across participants and in how far the resulting vectors were comparable in general, we calculated their overall agreement using the intra-class correlation coefficient (ICC). Additionally, we compared the ICC of the rotated coordinates to that of the original coordinates from before the coordinate system rotation. To make them comparable to the rotated coordinates, the original coordinates were also subjected to the same process of planar projection as described above, removing the *z*-axis.

## Results

### Intra-class Correlation Coefficient of Absolute Agreement

To test the reliability of OpenFace across so many different participants, we conducted an intra-class correlation coefficient (ICC) analysis. The ICC is a measure generally used to assess the agreement or consistency of multiple raters across different cases using continuous dependent variables. Given nothing but the OpenFace vectors, we would not have been able to conduct this analysis, but here comes into play the data obtained during manual annotation. Since we analyzed all participants twice, once with each approach, we were able to temporally map every OpenFace vector onto a grid label, allowing us to evaluate the extent to which the OpenFace vectors were similar across participants and grid labels. One way to conceive this is to imagine a scenario in which the participants were called into the lab in order to rate the 9 grid sections using their eye gazes. Imagine that, in each trial, they had been given a general direction to look in (the case, i.e. one of the nine gaze directions), and the specific location they looked at was their rating (the continuous dependent variable, i.e. the OpenFace vector). This was feasible, as our manual annotation of grid labels was determined to have a high inter-rater reliability (overall kappa value of 0.907), allowing us to be reasonably certain regarding the participants’ general gaze direction. For each participant, a mean vector was calculated for each gaze direction, so that, theoretically, there should have been 9 vectors (we excluded the gaze direction ‘0’ since it was more of a catch-all category for unusable gazes), as “ratings”, for each participant. In reality, however, a few participants did not look in all gaze directions, so that these vectors could not be included in the analysis.

The type of ICC we ran was a two-way random-effects model measuring absolute agreement with single measurements or, in other words, an ICC(2,1) model according to Koo and Li (16). Choosing the right ICC model was non-trivial, as our design proved to be unique in its interaction between raters (the participants) and the ratees (the gaze directions). The assumption made in the ICC(2,1) model is that each rater rates each ratee, which was not quite the case in our experiment, since some participants left out certain gaze directions. The other candidate model was the ICC(1,1), in which the assumption is made that a subgroup of raters rates a subgroup of gaze directions. Our design is situated somewhere between the two, in the sense that each rater/ participant could have rated each gaze direction, but it just so happened to be the case that some did not, meaning that most raters rated all gaze directions, while a few did not. Since we wanted to confidently generalize our results to all potential raters with the same characteristics as ours, we opted for the ICC(2,1) model. Incidentally, though, the ICC(1,1) model gave the same results as reported for the ICC(2,1) below.

We calculated an ICC with both the original, unrotated data, as well as the processed, rotated data in order to judge the degree to which the adjustments made rendered the coordinate systems more similar. It turned out, however, that the two types of data received the same ICC rating, for which reason the results reported below are representative of both.

ICC estimates and their 95% confidence intervals were calculated using R (23) and the R ‘psych’ package with its ICC() function, based on a single-ratings, absolute-agreement, 2-way mixed-effects model (i.e. ICC(2,1)). Since our vectors had two dimensions, *x* and *y*, we performed two separate ICC tests, one for each. The ICC(2,1) for the *x* dimension was estimated at 0.83 with 95% confidence interval = 0.71-0.94. As per Koo and Li (16), this result indicates a ‘moderate’ to ‘excellent’ agreement. The ICC(2,1) for the *y* dimension was estimated at 0.74 with 95% confidence interval = 0.58-0.90. This result indicates a ‘moderate’ to ‘good’ reliability (16).

Superficially, given the ‘moderate’ to ‘good’ ICC, the overall match between the different raters seems satisfactory, and one might conclude that OpenFace’s previously discussed tendency to underestimate the more extreme NVEMs is more or less systematic.

Moreover, the smaller range of motion in the analysis by OpenFace might very well have contributed to a lower score, since the vectors are more centralized than they should have been. The reason for this is that the ICC is sensitive to the overall range of the ratings, with the score increasing as the range increases. In other words, if the vectors had had a wider spread, i.e. a larger range, the ICC might have been higher in turn (20, p. 2471).

In order to further assess the compatibility of the OpenFace vectors with our manual coding grid, we turned to clustering.

### Reconstructing the Coding Grid from OpenFace

The final step in our analysis of the OpenFace data consisted in determining whether the coding grid could be reconstructed using clustering algorithms, viewing the clusters as equivalent to the coding grid sections. Out of the four algorithms used, *DBSCAN*, *k-means* (22), *k-medoids* and *k-medians* (21), only *k*-means provided consistent results between iterations that were also interpretable in terms of our coding grid.

For that reason the following discussion will focus on the results obtained via *k*-means exclusively, which do in fact show an emergence of a coding grid similar to the one that we utilized for the manual annotation.

**Figure 5.**
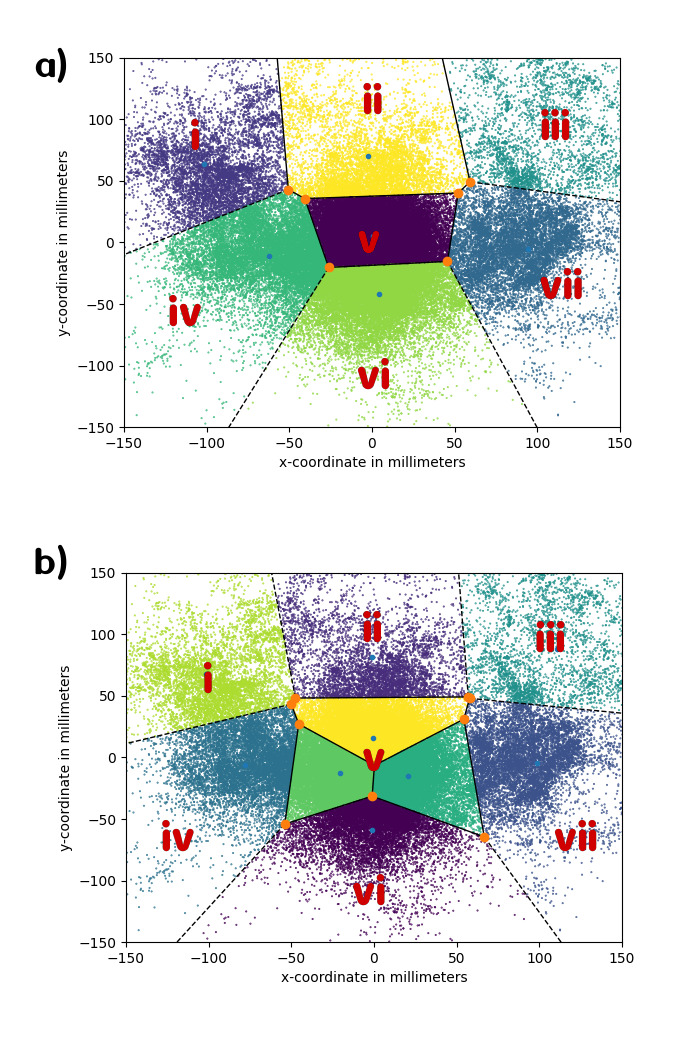
a) Graphical output of the *k*-means clustering algorithm with 7 clusters. One can observe that there are two mid-to-low peripheral sections combining the sections ‘4’ and ‘7’, as well as ‘6’ and ‘9’ from the manual coding grid. b) Graphical output of the *k*-means clustering algorithm with 9 clusters. Here, rather than differentiating the lower peripheral clusters into ‘4’ and ‘7’, and ‘6’ and ‘9’ as in the manual coding grid, two additional sections in the center area appear.

Since *k*-means takes as specification the amount of clusters it is supposed to derive from the given data set, and we did not want to presuppose our 9 coding grid sections, we ran the algorithm with all numbers ranging from ‘3’ through ‘10’. The sectioning most similar to the manual coding grid was obtained when the number of clusters was ‘7’ (see Fig. 5 a)).

Above 7, the center section of the coding grid became over-determined (see Fig. 5 b)), in the sense that multiple clusters occupied the space that was allocated to only one section in the original coding grid used for manual annotation. On the other hand, the mid-to-low peripheral sections from the manual coding grid remained merged into one.

Above 9, the additional clusters started to be appended to peripheral locations on the outside of the cluster structure, comprising only very few gazes. Since these new clusters proved to be uninterpretable with regards to the manual grid, we decided to exclude these clusterings from our analysis.

Below 7, the various sections start to merge into each other (see Appendix 3). At 6 clusters, the middle top section is divided up into the top left and top right ones, as well as the center section. At 5, the right-hand side of the grid has become one single cluster, while the center section starts opening up towards the top. At 4, the remaining clusters denote left, right as well as middle top and middle bottom. At 3, the whole data set is divided up horizontally into left, center and right, indicating a better horizontal resolution of NVEMs in our data set.

## Discussion

In this article, we have presented two approaches to analyzing NVEM (non-visual eye movement) data. The first approach employed manual annotations made by human annotators on the basis of a specific coding grid (see Fig. 1 a)), while the second approach employed automatic annotation with the open-source software OpenFace whose output is vector-based (see Fig. 1 b)). We compared the two approaches *via* one quantitative measure, the ICC (Intraclass Correlation Coefficient), as well as one qualitative measure, the reconstruction of the manual coding grid through the *k*-means algorithm.

The ‘moderate’ to ‘good’ ICC, gives some reason to believe that the two approaches provide converging data to a certain degree, and that a mapping from one representation to the other is feasible. Furthermore, in the case of the automatic annotations the ICC could have been reduced due to the systematic underestimation of extreme NVEMs, leading to a centralization of the whole data set. With the most extreme NVEMs being mapped towards the center, the potential deviations between participants could have been inadvertently smoothed out, yielding a lower ICC than appropriate.

Further evidence for some form of connection between the two representations (grid *vs*. vectors), although with small modifications to the coding grid, comes from the results of the *k*-means algorithm when applied to the OpenFace data. The emergent clusters divided up the data in ways that resembled the manual coding grid, however some unevenness remained. Either the lower peripheral sections merged with the mid peripheral sections (at 7 clusters) or the center section became over-determined (8 and 9 clusters). Interestingly, we also encountered difficulties in keeping distinct the mid and lower peripheral sections (i.e. ‘4’ and ‘7’, ‘6’ and ‘9’) during the manual annotation phase, stemming from frequent gazes towards the dividing lines between them. This, together with the 7 cluster reconstruction, gives reason to believe that the initial manual grid consisting of 9 sections might not categorize NVEMs accurately, and would need to be readjusted.

Moving to the cluster reconstructions with 8 and 9 clusters, the over-determination of the center section is another piece of evidence calling into question the 9-fold division of the manual coding grid.

This over-determination of the center section might have two reasons: first, it may be due to the predominance of recorded gazes towards the center of the screen, and a relative lack of gazes away from the screen, implying that with a more even gaze distribution, the clusters, too, would be distributed more evenly. Second, it might hint at a situation where gazes towards the center section actually have a more finely grained resolution, cognitively speaking, than we initially assumed in our manual grid. Perhaps, where exactly one’s non-visual gaze “falls” inside the center section, indicates a particular state of mind regarding episodic/semantic cognition.

Overall, the horizontal discrimination of gaze directions seemed to be more fine-grained than the vertical discrimination, something which is corroborated by both the higher ICC score for the *x*-dimension than the *y*-dimension, as well as the good horizontal fit of the *k*-means clustering. It appears to be a known phenomenon, that gaze analysis software boasts higher accuracy on the horizontal axis than on the vertical one (3). This is because of the reduced amount of pixels in the eye that are available for determining vertical movements, something which stems from the oval shape of the human eye.

On a related note, the human visual field is set up in such a way that we have a shorter eye movement range on the vertical axis than on the horizontal one (10). This reduced vertical range could be the reason for a lower ICC score in the vertical dimension, since the ICC decreases as the range of measurements decreases (20) [and see Results - Intra-class Correlation Coefficient of Absolute Agreement].

As of yet, it remains unclear whether it was because of the technical limitations that the ICC was lower for the *y*-component than for the *x*-component, or because of the higher impact that larger ranges of measurements have on the ICC. Further research is still needed to resolve this ambiguity.

In the same vein, the distortedness of our visual field, privileging the horizontal dimension, might also lead to a higher propensity to gaze horizontally, and thus be the reason for the better horizontal resolution in clustering we obtained. Thus, in the most coarse-grained, 3 *k*-means clustering, we were left with a purely horizontal clustering (left, middle, right). Perhaps, due to the anatomy of our eyes, we have better cognitive resolution on the horizontal axis. In support of this, higher cognitive resolution in the horizontal dimension has indeed been found in a study investigating visuo-spatial short-term memory (5).

The process of analyzing the data may have been more straight-forward if we had designed our experiment from the outset with the intention of analyzing it with OpenFace. Here, we applied this mode of analysis in a *post-hoc*, exploratory manner, taking extra steps in order to address some of the incompatibilities in the original experiment, such as not having a dedicated calibration phase designed to enable better alignment of the OpenFace vectors between the participants. Furthermore, future research could experiment with camera placements below the screen in order to mimic conventional eye-tracking setups more closely and potentially boost OpenFace performance.

Interestingly, the rotation step we performed made no difference in terms of the convergence between manual coding grid and vectors. This is indicated by the equal ICC scores for rotated vector data *vs*. original vector data and casts doubts on whether such a step would have been necessary. The reason for this is most likely that the extent of this *post-hoc* alignment was too insignificant to have any impact on the ICC calculation. Whether this can be assumed for all future research of this kind is unclear however, as the exact experimental set-up is subject to contingencies. Also, even though the coordinate rotation may not have any significant bearing on the ICC calculation, subsequent analyses might well benefit from it. Therefore, in order to eliminate this ambiguity, future experimenters looking to utilize OpenFace or similar software would be advised to devise some kind of calibration phase, one that does not alert the participants as to the study’s focus on eye-movements.

Having established both a moderate to good reliability for each method in quantitative terms (*via* the ICC and Cohen’s kappa), and internal validity for the coding grid in qualitative terms (*via* the *k*-means clustering), a separate quantitative measure of performance similarity between the two methods would further inform their compatibility. At this point, compatibility remains to be inferred from the visual similarity of the *k*-means clusters with the coding grid layout. But in a subsequent step, one might also compare their respective performance when subjected to the same type of analysis. For this purpose, since categorical grid sections and continuous vectors cannot serve as input for one and the same analysis, the representations must be assimilated, which is achieved by our *k-*means clustering. To conduct a joint analysis then, following a suggestion received from an anonymous reviewer, various AOI (area-of-interest) analyses could be applied to both our coding grid annotations as well as our *k*-means clusters derived from OpenFace vectors. The discrepancy of the results could serve as a first quantitative measure of the degree to which they are able to perform comparably. Future research will be needed to accomplish this aim.

Deciding whether one approach, manual coding grid or automatic OpenFace analysis, is superior to the other should in part depend on the research question one is investigating, as well as the available resources one has access to. For instance, on the one hand, we have used the manual annotations to show that speech rate while producing episodic past, episodic future, or semantic descriptions varied significantly across the grid categories (24), indicating differential coordination of (non-visual) eye gaze and language production. On the other hand, we have made use of OpenFace’s vectors by applying velocity- and dispersion-based algorithms on the data , allowing us to extract saccade and fixation lengths (14; 15, see also below).

The coding grid approach is labor-intensive, but intuitive and yields consistent results across raters. Furthermore, it allows the eye gazes to be annotated in a way that includes a dimension of meaning which is difficult to reconstruct in the automatic approach. For instance, the annotators would be able to identify the target direction of certain gazes at the point at which the participant first started to move their eyes towards it. In how far this goal-directedness can be inferred from OpenFace’s data is unclear. Whether this dimension of meaning is desirable in the first place, depends on the specific research intent. Following recent trends in 4E cognition, perception is always already meaningful (26), which gives reason to consider the possibility that a manual annotation can capture particular aspects of NVEMs more faithfully. Finally, it is unclear whether the nine-fold division of the coding grid is actually appropriate.

OpenFace, on the other hand, while less labor-intensive, does require some proficiency in programming and data visualization techniques. Moreover, the OpenFace documentation, while having grown in completeness over the years, is still rather broad at the time of writing, so that finding answers to more specific questions is not always easy.

OpenFace also offers a higher temporal, as well as spatial, resolution regarding the eye gaze annotations. Despite this, we cannot reliably conclude that OpenFace surpasses the manual approach concerning the precision in the spatial dimension, due to its systematic underestimation of extreme gazes, discussed at the end of the section Annotation methods - Automatic annotation with OpenFace. This is the case especially since extreme gazes are of high interest to the study of NVEMs. Future technological improvements might however resolve this problem, as eye gaze estimation becomes more accurate.

Additionally, there may be some less obvious biases in OpenFace. For instance, it would seem as though OpenFace calculates its world coordinates, which form the basis of its vector representations, on the assumption that the distance between a person’s eyes is exactly the “average” distance of 65 mm (2). Of course, the term “average”, here, is laden with presuppositions, something to be kept in mind. Also, the training data on which OpenFace was trained seems to struggle with capturing the faces of children and people of Asian descent (11). However, this can be remedied by retraining OpenFace with a more appropriate data set if the circumstances require it.

Nonetheless, OpenFace has performed very well in comparative studies with other eye/face-tracking software (1, p. 60, TABLE I), suggesting that it is the most likely contemporary gaze analysis software to yield good results. In light of the emergence of clusterings reminiscent of our coding grid from the *k*-means algorithm, there does appear to be a high enough accuracy and resolution to make it suitable for certain research scenarios in which the capacity for labor is low. It is also worth noting that OpenFace offers a host of additional information, such as “Facial Action Coding”, and various facial landmarks that may be put to valuable use.

All in all, our chief finding is best described as providing two distinct, but potentially converging approaches to the study of NVEMs. The exact degree of convergence remains an open question, however.

For now, there are few open-source programs that focus on non-intrusive eye gaze estimation specifically, which naturally hinders the proper investigation of NVEMs. We hope that the future will provide more sophisticated tools in this area, and that the procedures documented here will be of help in elucidating the nature of NVEMs and in what way they are associated with our cognitive processes. For example, Kock (14) (see also 15) used the automatically annotated data from OpenFace to calculate fixation and saccade lengths based on algorithms identifying velocity and dispersion thresholds (I-VT and I-DT), respectively. Their distribution across (past and future) episodic narration *vs*. semantic description were calculated. Kock did not find any differences between the three conditions – hinting at rather similar general processes underpinning these cognitive systems – at least as indicated by eye-gaze features. Future research based on the present assessments of alternative methods annotating NVEMs, may elucidate this connection further.

### Ethics and Conflict of Interest

We declare that the contents of the article are in agreement with the ethics described in http://biblio.unibe.ch/portale/elibrary/BOP/jemr/ethics.html and that there is no conflict of interest regarding the publication of this paper.

### Acknowledgements

We acknowledge support by Deutsche Forschungsgemeinschaft (DFG) and Open Access Publishing Fund of Osnabrück University.

We wish to thank Jana Kernos for designing the original experiment, and Mirko Kirchhoff for offering helpful remarks.

## Figures and Tables

**Figure 6.**
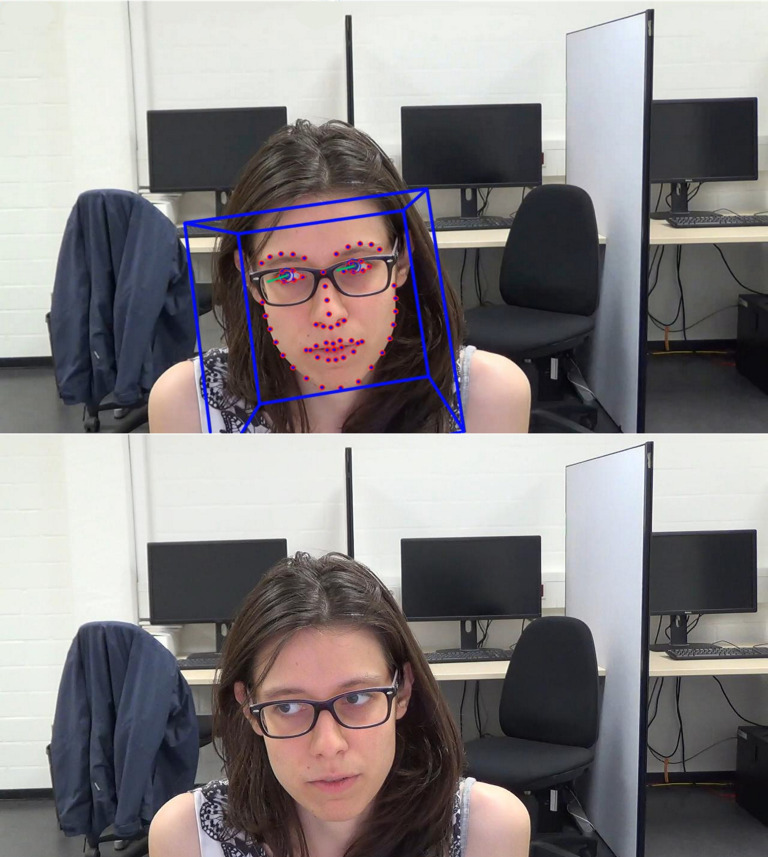
Example of the consistent underestimation of gaze angles on the part of OpenFace. Top: Visual output of OpenFace including the vectors estimating the participant’s gaze angle. Bottom: original video footage. In this case, while human observers rate the participant’s gaze angle as ‘4’, the OpenFace gaze vectors are oriented in a direction corresponding to ‘7’.

**Figure 7.**
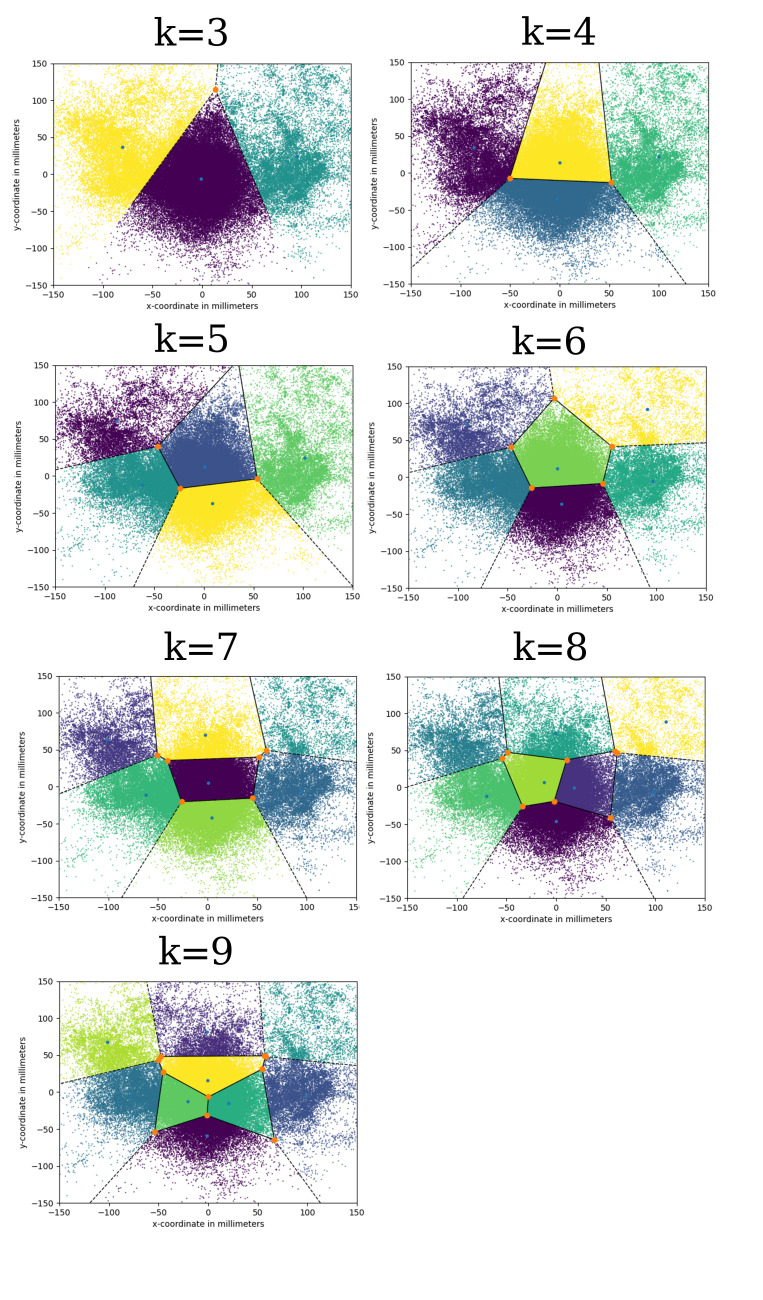
Graphical output of the *k*-means clustering algorithm with 3 to 9 clusters.

## Appendix

### Appendix 1: Experimental Method

The aim of the experiment was to investigate whether NVEMs differ in terms of memory conditions (Kernos, 2019; Stelter, 2019). To do this, participants’ faces were recorded while they verbally described either the sequential, episodic unfolding of situations in the past or in the future, or the semantic description of certain objects. Please note that, for our purposes of analyzing the different ways of annotating the data, the exact experimental design is secondary and is listed here for the sake of illustration and completion only.

**Participants**

The 13 participants were students at the University of Osnabrück. They all had normal, or corrected vision. Upon completion of the experiment, they were licensed to receive course credit.

**Design**

Participants were asked to describe everyday objects in three conditions: (1) semantically, explaining what the object is; (2) narrating an episodic memory involving the object; and (3) narrating a future episode involving the object. The phrasing of the tasks for (1) was “Describe what X is.”. For (2) and (3) it was “Describe a situation with X from last week/within the next week.”. X was an object like “a plant”. Additionally, at the beginning of each trial, one of three types of images appeared in the center of the screen. The first type was an image of the object from the corresponding condition, while the second and third were a male and female face respectively. These were not relevant for our analysis, though.

**Materials**

The prompts at the beginning of each trial, specifying either the situation or the object to be described, were read by a computer-generated voice. There were 12 different stimuli in total. Every participant completed all 12 trials in pseudo-randomized order.

**Procedure**

After signing the consent form, the participants were seated in front of a computer screen. A video camera was installed behind and slightly above the screen, frontally capturing the participant’s reactions. A frontal perspective was most straightforward for human annotators, as it resembled day-to-day interactions and provided the most complete view of the participant’s face. At the beginning of each trial, the subjects were informed about the condition and object and one of the three types of images appeared in the center of the screen. Then the participant verbally responded. There was no time limit with regards to either the participant’s planning of their response, or the completion of each trial. Once the participant felt that they had said enough, they proceeded via the press of a button. Afterwards, they answered three questions concerning their experience of the process of answering the trial.
